# Comorbidities of Patients with Functional Somatic Syndromes Before, During and After First Diagnosis: A Population-based Study using Bavarian Routine Data

**DOI:** 10.1038/s41598-020-66685-4

**Published:** 2020-06-17

**Authors:** Ewan Donnachie, Antonius Schneider, Paul Enck

**Affiliations:** 1 Department of Internal Medicine VI: Psychosomatic Medicine and Psychotherapy, University Hospital Tübingen, Germany and TUM School of Medicine, Institute of General Practice and Health Services Research, Technical University of Munich, Munich, Germany; 2 TUM School of Medicine, Institute of General Practice and Health Services Research, Technical University of Munich, Munich, Germany; 3 Department of Internal Medicine VI: Psychosomatic Medicine and Psychotherapy, University Hospital Tübingen, Tübingen, Germany

**Keywords:** Irritable bowel syndrome, Psychiatric disorders, Gastroenteritis, Infection, Comorbidities

## Abstract

Functional somatic syndromes (FSS) are characterised by the presence of one or multiple chronic symptoms that cannot be attributed to a known somatic disease. They are thought to arise though a complex interaction of biological and psychosocial factors, but it is unclear whether they share a common aetiology. One hypothesis supported by recent studies is that the FSS are postinfectious disorders, as is widely recognised for a subset of patients with irritable bowel syndrome. Our study used claims data submitted by office-based physicians to compare groups of patients with different FSS in the five years before and after the point of first diagnosis. Even five years prior to diagnosis, FSS patients consulted more frequently for a range of psychological and somatic conditions than did controls. Following diagnosis, consultation rates increased further and remained persistently high. Five years after diagnosis, between 34% (somatization disorder) and 66% (fibromyalgia) of patients were still being treated for the condition. Both prior gastrointestinal and upper-respiratory infection were associated with an increased risk of developing an FSS. We therefore recommend that patients at risk should be identified at an early stage and the underlying psychosocial and somatic issues addressed to prevent progression of the condition.

## Introduction

Functional somatic syndromes (FSS) are typically characterised by the presence of one or multiple chronic symptoms that cannot be attributed to a known somatic disease. The prevalence is high and estimated to be up to 34.8% in primary care^1^. The term encompasses a range of syndromes that are diagnosed according to consensus-based criteria with respect to the predominant symptom or symptoms^2,3^. For example, persistent gastrointestinal complaints may be labelled as irritable bowel syndrome (IBS), fatigue as chronic fatigue syndrome (CFS), and widespread pain as fibromyalgia.

Significant overlap exists between the FSS with respect to the symptoms reported, with a substantial proportion of patients meeting the criteria for more than one FSS condition^3^. There are striking similarities with respect to the proposed aetiology, pathophysiological mechanisms, psychological comorbidity, treatment response and patient characteristics^4^. Some researchers therefore propose that these syndromes are best understood as one single disease entity with varying phenotypes, as opposed to many distinct diseases^5^. However, neither the “lumping” nor the “splitting” approach seems to accommodate fully the considerable heterogeneity that is observed both within and between the individual syndromes^3^. The various diagnostic labels are therefore best viewed as working diagnoses, combining a clinically useful description of the syndrome with a tentative aetiological model.

Several possibly interacting mechanisms have been proposed to explain why some people develop chronic functional conditions. Of particular interest are the roles of the central nervous system, the immune system and the microbiome. One important hypothesis posits that the FSS, or a subset thereof, are post-infectious syndromes. While the occurrence of chronic symptoms following viral or bacterial infection has long been documented, the role of infection in the pathogenesis of the FSS remains subject to debate^6,7^. There is convincing evidence that gastrointestinal infection is instrumental in the onset of IBS^8^. Likewise, CFS has been associated with acute Epstein-Barr infection^9^. A number of studies have further demonstrated that gastrointestinal infection increases the risk of CFS almost as much as it does IBS^10–15^. Little evidence exists as to whether this association holds for other functional syndromes such as tension headache or fibromyalgia. Such a global effect is however conceivable, as gastrointestinal infection may lead, for example, to a disturbance of the gut-brain axis, to autoimmune reactions, or to dysbiosis of the microbiome.

Both functional and postinfectious syndromes are generally viewed within a bio-psychosocial framework, with the propensity to suffer long-term distress associated with certain psychological traits^16–18^. They often occur alongside psychological illnesses such as depression, anxiety and stress reaction disorder. However, the interaction of biological processes and psychosocial factors in the development and perpetuation of FSS is poorly understood, as is the relation of postinfectious cases to cases without any known infectious prequel. Similarly, it is unclear why, for example, one patient might present with postinfectious IBS and another with postinfectious CFS.

### Objective

In this context, it seems important to determine how the various FSS present in primary care over an extended period of time, both before and after first diagnosis. Most studies of functional somatic syndromes in a primary care setting are however cross-sectional in nature or have only a short followup period, often with little or no differentiation between newly diagnosed and long-term cases. Retrospective studies of patients with functional conditions often suffer from recall bias, potentially inflating the importance of prior events or comorbidities. We therefore utilise longitudinal claims data to provide a new perspective on the FSS, observing patients in the five years before and after their first diagnosis in ambulatory care. Our specific research questions were:

1. How do different FSS conditions compare with respect to the distribution of age and sex at first diagnosis?
2. What proportion of patients remain in treatment for the condition 1, 2 and 5 years post-incidence?
3. How do the rates of consultation for psychological disorder, somatic symptoms and infection develop in the period before and after first diagnosis of a FSS?
4. Is prior infection in general and gastrointestinal infection in particular associated with the onset of FSS?

## Methods

### Study data

We analyse anonymous claims data held by the Bavarian Association of Statutory Health Insurance Physicians (German: Kassenärztliche Vereinigung Bayerns, KVB). The data cover approximately 85% of the population of Bavaria (2010: 10.4 million people with statutory health insurance) and are submitted by licensed general practitioners, office-based specialists and psychotherapists primarily for the purpose of remuneration. All diagnoses relevant to the treatment episode are recorded on a quarterly basis using the German modification of the ICD-10 classification. The database allocates a unique and persistent pseudonym to each patient, removing all personally identifying information (name, insurance number, exact date of birth and address etc.) in order to protect the identity of the patients. The study was conducted according to the German guideline “Good Practice of Secondary Data Analysis^19^.

### Cohort

The cohort consists of patients aged between 18 and 50 years with first recorded diagnosis of one of the following functional somatic syndromes in the year 2010: chronic fatigue syndrome (CFS), irritable bowel syndrome (IBS), other functional intestinal disorders (FID), fibromyalgia syndrome (FMS), tension headache (TH) and somatoform disorder (SD). The underlying ICD-10-GM codes are provided as supplementary information. Patients were included only if the diagnosis was repeated in a subsequent quarterly period. Patients were excluded if observed for less than two years prior to or following first diagnosis, thus ensuring an adequate observation.

In order to ensure comparability of the groups, all FSS patients were weighted such that the distribution of age, sex and district of residence matched that of the Bavarian population aged between 18 and 50 in the year 2010. A control group was drawn from the pool of patients with no recorded FSS diagnosis and meeting the same inclusion and exclusion criteria. The sample was stratified by age, gender and district of residence, with the number in each group chosen to be representative of the Bavarian population. The desired size of the control group was set to match the total number of FSS patients, with the exact size allowed to deviate to ensure proportionality of the strata. We thus generated six FSS groups and one control group.

### Statistical analysis

The cohort was first described using summary statistics and histograms to investigate the size and structure of the seven cohort subgroups.

To measure the persistence of the diagnosis, Kaplan-Meier estimators were applied to estimate the time between the first and last recorded diagnosis of the index FSS^20^. This was used to calculate the proportion of patients still in treatment for the condition after 1, 2 and 5 years. Patients were considered to be censored (i.e. with the diagnosis persisting until the end of the observation period) if the FSS diagnosis was recorded in the final year of that patient’s observation. To investigate possible diagnosis switching, the analysis was repeated to consider the time between first diagnosis and the last record of any FSS diagnosis.

For each quarterly period in the five years before and after initial diagnosis, the proportion of patients in each subgroup consulting for a range of somatic and psychological diagnoses was calculated. The resulting consultation rates provide an indication of the intensity of treatment during a given time period. Patients were included in this calculation only within the period they were observable in the data. In order to highlight the long-term pattern, the rates were smoothed using cubic regression spines and visualised, with the quarter of first diagnosis omitted.

In order to quantify the association between the development of a FSS and a record of prior psychological disorder or gastrointestinal infection, odds ratios were calculated by means of logistic regression, controlling for the age group and sex of the patient. As gastroenteritis is very common and an infectious origin often not clarified, cases of non-specific gastroenteritis were considered alongside those cases with confirmed infectious aetiology. Upper respiratory tract infections was included as further comparator diagnosis, allowing the specificity of the effect to be assessed.

Statistical analysis was performed using the R environment for statistical programming (Version 3.4.2), using the survival package to calculate the Kaplan-Meier estimators, the mgcv package to smooth the consultation rates and the ICD10gm package to utilise the ICD-10-GM metadata^21–24^.

### Ethics approval and consent to participate

In line with the German guideline for research conducted using secondary health care information, ethics approval and patient consent are not required for studies based solely on anonymised claims data^19^. The study was conducted under strict data protection restrictions as mandated by the data holder (Bavarian Association of Statutory Health Insurance Physicians).

## Results

**Figure 1:**
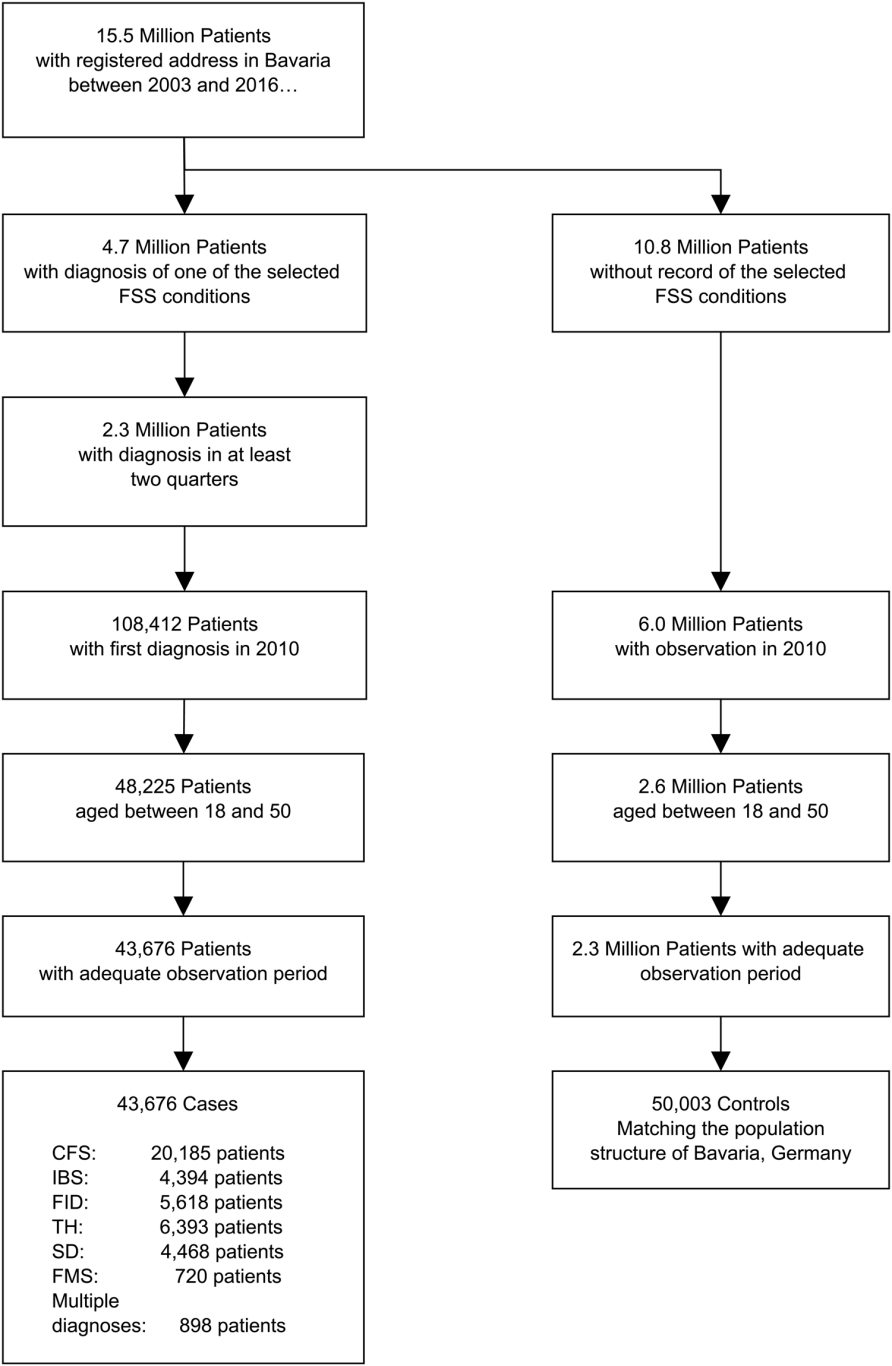
Schematic diagram of the study flow.

Figure 1 illustrates the selection of the study cohort. After applying the inclusion and exclusion criteria, 43,676 patients were identified with first diagnosis of a defined FSS condition in the year 2010. The subgroups ranged in size from 20,185 patients (Chronic Fatigue Syndrome) to 720 patients (Fibromyalgia Syndrome). 898 patients were coded with two different FSS diagnoses in the same initial quarter and were thus included in a “multiple FSS” group.

The FSS cases were complemented by 50,003 control patients with no record of these FSS conditions. The cohort therefore consists of 93,679 patients.

### Description of the cohort

Table 1 provide a basic summary of the cohort at the time of first diagnosis (cases) or time of inclusion (controls). Cases were on average 35.1 years old (controls: 34.8) and 67.6% were female (controls: 49.3%). The mean observation period for cases was 25.7 quarters before first diagnosis and 28.5 quarters following diagnosis, compared to 24.6 quarters and 26.8 quarters, respectively, for controls. 91.1% of cases and 90.1% of controls were observed for at least five years prior to first diagnosis, with 96.1% or cases and 94.0% of controls followed up for at least five years post diagnosis.

**Table 1:** Description of the cohort at time of first diagnosis.

Group	N	Age		Gender	Observation min. 5 years		Mean observation time	
		mean	sd	female (%)	pre (%)	post (%)	pre (quarters)	post (quarters)
Control	50,003	34.8	9.4	49.3	90.1	94.0	24.6	26.8
All cases	43,676	35.0	9.4	67.4	91.1	96.1	25.6	28.5
CFS	20,185	35.8	8.9	68.8	91.2	96.1	25.6	28.5
IBS	4,394	33.8	9.7	64.3	91.0	96.2	25.7	28.4
FID	5,618	34.0	9.7	67.4	90.7	96.5	25.6	28.6
FMS	720	41.4	7.0	87.1	92.5	96.8	26.1	28.8
TH	6,393	32.7	9.9	67.0	90.6	96.2	25.5	28.5
SD	5,468	36.2	9.1	62.3	91.6	95.9	25.7	28.4
Multiple FSS	898	35.5	9.1	69.6	91.1	95.7	25.6	28.4

**Figure 2:**
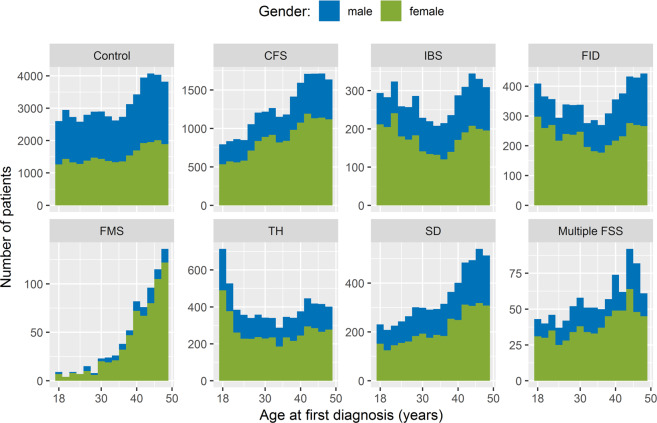
Age distribution of cases and controls at the time of first diagnosis. Each histogram displays a subgroup of the cohort and is stacked to show the relative proportions of male and female patients for each two-year age interval.

Figure 2 shows clear differences between the conditions with respect to the distribution of age and sex. In particular, whereas FMS is found predominantly in female patients over the age of 40 years, tension headache appears to peak in young adults between the ages of 18 and 25.

### Persistence of the diagnosis

**Figure 3:**
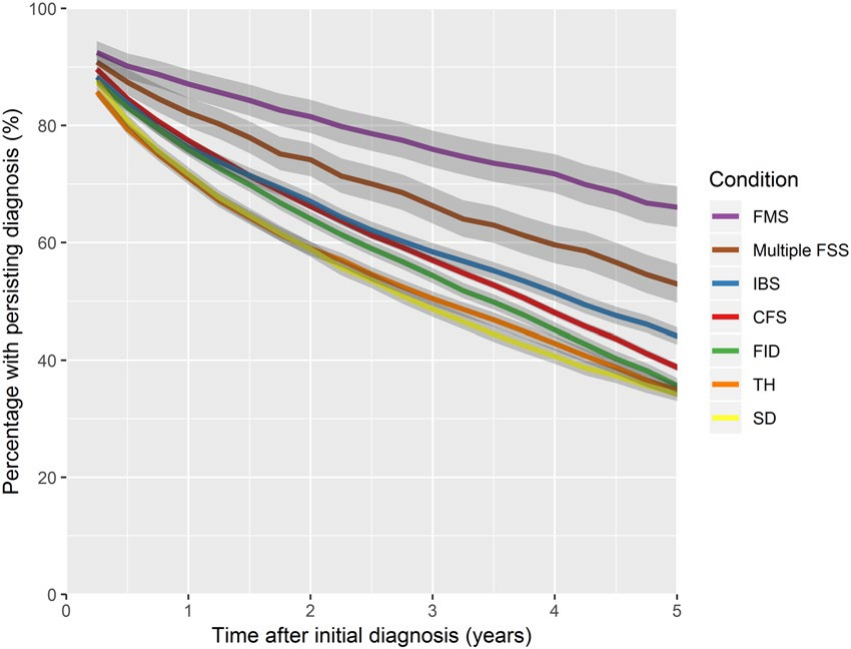
Kaplan-Meier estimation of the time between the first and last recorded diagnosis of the FSS conditions.

Figure 3 displays a Kaplan-Meier estimation of the time between the first and last recorded diagnosis of the FSS conditions. A duration of at least two quarters is ensured by design. Two years after the initial diagnosis, between 59% (TH, SD) and 82% (FMS) of patients have a continued record of the condition. The proportion of cases with any FSS diagnosis at two years follow-up is higher, ranging between 70% (TH) and 88% (FMS). After five years, the corresponding proportions with diagnosis of the same condition were 34% (SD) and 66% (FMS), with between 46% (CFS, TH, SD) and 73% (FMS) having a record of any FSS condition. Further details are provided as supplementary information. The persistence of the diagnosis varies significantly between FSS groups (log-rank test: p < 0.001).

### Consultation for selected diagnoses

**Figure 4:**
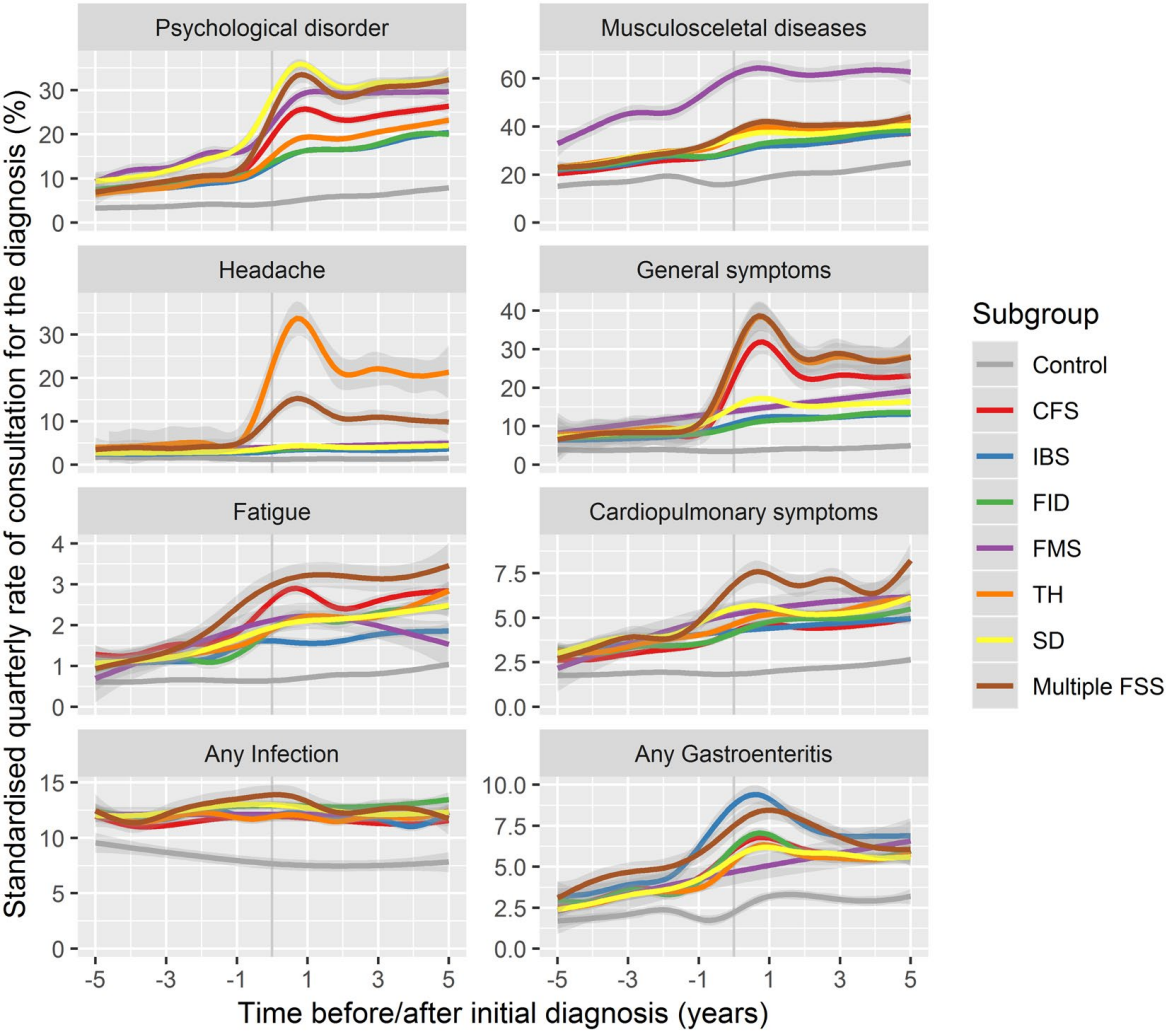
Smoothed estimation of the percentage of patients consulting with various diagnoses in the 20 quarters before and after first FSS diagnosis.

Figure 4 displays the proportion of patients consulting a physician for selected diagnoses per quarterly period. In order to highlight the long-term trend, the results for the individual quarters are smoothed and the quarter of incidence is ignored. More extensive results are presented as supplementary material and important results outlined in the following subsections.

### Psychological disorder

Five years before first diagnosis of a FSS, the rate of consultation for psychological disorder is more than twice as high among future cases as compared with control patients. Over the entire pre-incidence period, between 25% (IBS) and 51% (FMS) of cases received a diagnosis of depression, anxiety or stress reaction disorder in at least one quarter, as opposed to 13% of controls. After standardising to the age and sex distribution of the population, no substantial differences are visible in the consultation rates of the six FSS groups, with approximately 10% of patients receiving a diagnosis in a given quarterly period, compared with under 5% of controls.

In the two years before and after the first diagnosis, the rate of consultation rises sharply in all FSS groups but only gradually in the control group (e.g. due to increasing age and/or an evolving health system). Differences emerge between the FSS conditions, with approximately 30% of patients with FMS, somatoform disorder and patients with multiple conditions receiving a diagnosis of depression, anxiety or stress reaction disorder in a given quarterly period, compared with approximately 20% of those with IBS, functional intestinal disorder and tension headache. This high rate of treatment for psychological conditions is sustained throughout the five year follow-up period.

### General symptoms

Before the initial diagnosis of a FSS, all groups present more frequently with back pain, general complaints (including fatigue, headache, dizziness and unspecific cognitive symptoms), gastrointestinal symptoms and cardiopulmonary symptoms than do controls.

Diseases of the musculosceletal system (ICD-10 Chapter XIII) and, in particular, back pain, are the most frequently observed of the diagnoses among those considered. After adjustment for age and sex, patients with fibromyalgia display a substantially higher rate of consultation than do other cases or controls. Five years before the initial diagnosis, approximately 35–40% of the fibromyalgia patients consult for musculosceletal diagnoses in any given quarter, after standardisation to the age and sex distribution of Bavaria. This compares with 20–25% in other case groups and 18% in controls.

While the FSS diagnoses differ in terms of the prominent features, they all exhibit increased rates of consultation with general symptoms both before and, to a greater extent, after first diagnosis of the leading FSS, as compared to the controls.

### Infection

FSS cases are more often treated for infectious diseases than are controls. Prior to first diagnosis, between 79.0% and 81.4% of the FSS groups had a record of an infectious disease, as compared with 68.9% of controls. Both for infectious diagnoses as a whole and for upper respiratory infections, there is no substantial change in treatment intensity over the course of the observation.

Gastroenteritis was recorded for between 39.5% (TH) and 49.2% (multiple FSS) of cases prior to diagnosis, as compared with 27.0% of controls. A specific infectious aetiology (i.e. a laboratory confirmed diagnosis) was recorded for between 6.1% (FMS) and 11.4% (IBS) of patients, as compared with 4.7% of controls. Whereas controls exhibit a gradual increase in the intensity of treatment for gastroenteritis over time, there is a marked increase in all FSS groups in the year before first diagnosis, with the intensity remaining constant at approximately 5–7% of patients per quarter for the entire post-FSS observation period (controls: 3%).

### Risk factors

**Figure 5:**
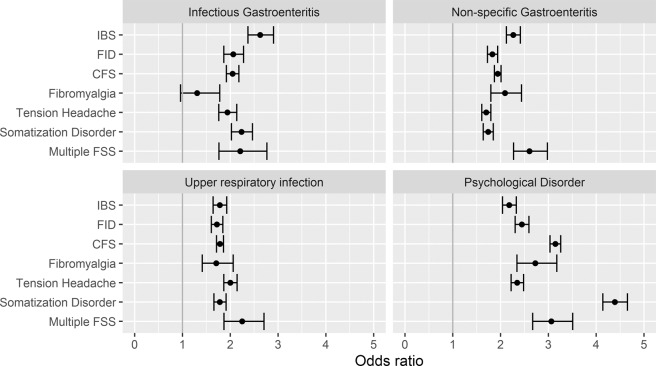
Odds ratios for the diagnosis of prior gastrointestinal infection, gastrointestinal symptoms, psychological disorder and unspecific somatic symptoms in dependence on functional somatic syndrome, age group and gender.

Figure 5 and Table 2 display the odds ratio for the incidence of a FSS condition with respect to the following prior diagnoses: non-specific and specific infectious gastroenteritis, psychological disorder and upper respiratory infection. It may be seen that consultation for either infectious gastroenteritis or psychological disorder is a significant predictor for all FSS conditions. For prior infectious gastroenteritis, the odds ratios range between 1.31 (fibromyalgia) and 2.62 (IBS) For prior psychological disorder, the odds ratios range between 2.18 (IBS) and 4.39 (somatization disorder). That similar associations are present for upper respiratory infection indicates that these results are not specific to the site of the infection.

**Table 2:** Odds ratios for the association between various exposures and the subsequent diagnosis of a FSS.

FSS Group	Infectious Gastroenteritis	Non-specific Gastroenteritis	Upper respiratory infection	Psychological Disorder
CFS	2.05 (1.92, 2.18)	1.94 (1.87, 2.01)	1.78 (1.71, 1.86)	3.14 (3.03, 3.26)
Fibromyalgia	1.31 (0.96, 1.78)	2.09 (1.79, 2.44)	1.71 (1.41, 2.06)	2.73 (2.34, 3.18)
FID	2.06 (1.87, 2.28)	1.83 (1.72, 1.94)	1.72 (1.60, 1.84)	2.45 (2.31, 2.59)
IBS	2.62 (2.37, 2.91)	2.26 (2.12, 2.41)	1.78 (1.64, 1.93)	2.18 (2.04, 2.33)
Multiple FSS	2.21 (1.76, 2.76)	2.60 (2.27, 2.98)	2.25 (1.87, 2.70)	3.06 (2.67, 3.51)
Somatization Disorder	2.23 (2.02, 2.46)	1.74 (1.64, 1.85)	1.78 (1.66, 1.91)	4.39 (4.14, 4.65)
Tension Headache	1.94 (1.76, 2.14)	1.70 (1.61, 1.80)	2.00 (1.87, 2.14)	2.35 (2.22, 2.48)

## Discussion

To our knowledge, the present study is the first to observe a cohort of patients in primary care in the five years before and after the first diagnosis of a FSS. By comparing the profiles of different FSS with respect to the diagnoses recorded by their physicians, we provide a new perspective on these complex and interrelated conditions. Our main results are a markedly increased prevalence of psychological disorder, somatic symptoms and infection that is present at least five years before the first FSS diagnosis, intensifies further around the time of first diagnosis, and persists in a substantial proportion of the patients.

Similar to a previous study using the same study database^10^, we find a significant association between both prior psychological disorder and gastrointestinal infection and the development of FSS. While the present case-control study provides weaker evidence for a causal link than the previous cohort study, the observed associations are comparable and are extended to patients with tension headache, fibromyalgia and somatoform disorder. Of particular interest is the increased occurrence of gastroenteritis immediately before first diagnosis and persisting throughout the follow-up period. Taken together, our results demonstrate that the role of gastrointestinal infection is by no means specific to IBS. This is consistent with the “biology first” hypothesis formulated in response to explain a variety of similar research findings^7^, although our epidemiological data might reflect a correlative rather than a causal relationship.

The treatment history of the patients reveals similarities between the syndromes investigated. Patients from each FSS group exhibit a higher than average treatment intensity for the symptoms characterising other FSS groups. For example, all FSS groups exhibit an increased administrative prevalence of musculoscelatal symptoms such as back pain. Similar overlap has been noted in a variety of studies in different clinical settings^3^. For example, Fink and colleagues used the WHO’s schedules for clinical assessment in neuropsychiatry (SCAN) to screen for 76 distinct functional symptoms in a group of 978 patients, finding that the median number of distinct symptoms was five^25^. Among patients with IBS, a systematic review by Whitehead and colleagues found a high prevalence of somatic comorbidity, with 49% of patients meeting the criteria for fibromyalgia and 51% for chronic fatigue syndrome^26^. They considered the evidence for a common somatization disorder to be weak, instead arguing that psychological factors provided a common link between the various functional syndromes. A register-based study by Petersen and colleagues found a high burden of physical comorbidity among FSS patients as compared with control patients^27^. Physician-led telephone interviews among participants of the Danish Study of Functional Disorders (DanFunD) found high overlap between five different FSS disorders, observing 20 out of 26 possible combinations^28^.

Differences between the FSS are seen primarily in the strong expression of a leading symptom, for example gastrointestinal complaints for IBS, headache for tension headache and musculosceletal complaints for fibromyalgia syndrome. It should however be noted that the physician-recorded diagnoses analysed reflect an often selective and chaotic description of the complaints as perceived and reported by the patient^29^. On the other hand, the patients’ selection of medical subspecialty to consult or, in some healthcare systems, the subspecialty referred to by the primary care physician for further diagnosis, may determine the final diagnosis (e.g. IBS if a gastroenterologist is consulted, or chronic pelvic pain syndrome if seen by a gynaecologist or urologist)^5^. The observed predominance of certain symptoms is the product of a complex decision-making process both on the part of the patient and of the physician^30^. As observed in the routinely collected data, these leading symptoms may be viewed as an inherent part of the disease experience but do not necessarily imply the presence of distinct diseases or phenotypes. With FMS in particular, the comparison of raw and standardised results would suggest that the predominant symptoms are partially an expression of the biological, psychological and social status of an older, mostly female patient group. The prevalence of FMS is considerably lower than that of CFS, which might be explained by a reluctance on the part of physicians to label patients with a diagnosis of FMS, which is controversial in German primary care^31^. Further research is however required to better understand the aetiological relationship between the predominant and secondary symptoms.

Figure 1 shows that less than half of patients receiving a secured diagnosis of the specified functional syndromes consult for the same condition in a second quarterly period. It is unclear to what extent such patients were coded in error (e.g. acute gastrointestinal symptoms coded as functional intestinal disorder) and to what extent patients decide not to seek, or do not require, further treatment for an existing condition. For example, the provision of self-help information has been shown to improve the quality of life of IBS patients^32^, thus reducing the need for further consultation. Additionally, the Gemran FSS guideline advocates for restraint in diagnostic investigations when somatoform disorders are suspected^33^. Of those patients whose FSS diagnosis is confirmed in two quarters, there is a moderately high persistence, with between one third and two thirds of patients receiving the same diagnosis after five years. Relatively few patients transition from one FSS group to another; patients may, instead switch from one gastrointestinal phenotype, IBS, to another phenotype, functional dyspepsia^34,35^. Of note is that approximately 20% of patients from other FSS groups are diagnosed with CFS during follow-up, as compared with only 1.4% of controls.

With respect to the pathogenesis of the FSS, recent research has identified an alteration of the hypothalamic-pituitary-adrenal (HPA) axis activity in patients with both depression and somatization^36^, and also in patients with anxiety disorder^37,38^. Epidemiological studies have demonstrated their close relationship^39^. It might be speculated that concomitant disturbances of cortisol levels and adrenergic balances might not only increase symptom perception^36^, but also contribute to physiological impairments^40^. Beyond this, it was shown that gastrointestinal infections might contribute to alteration of subjective wellbeing by modification of the brain-gut-microbiota axis^7,41,42^. It is not possible to draw causal conclusions with our routine data analysis. However, the course of the diseases might reflect the impact of these neuro-endocrinological disturbances before the final FSS diagnoses were coded by the physician. Patients with subsequent FSS might encounter their physician more frequently due to enhanced susceptibility of bodily symptoms as delineated above. This might also explain why odds ratios were increased in respiratory tract infections.

### Strengths and limitations

A major strength of the present study is the analysis of longitudinal claims data, allowing the retrospective observation of anonymised patients over a period of more than 10 years. This enables the identification of a large cohort of newly incident patients with different functional diagnoses, who may be compared on an equal basis. The data cover 85% of the population of Bavaria, Germany, and therefore more representative than studies including, for example, patients in tertiary care or those covered by selected health insurance companies. Unlike most primary studies, prior co-morbidities may be assessed without the problem of recall bias. The changes in diagnostic patterns observed around the time of first diagnosis provide more robust insight than a cross-sectional analysis.

The use of routinely collected claims data may also be viewed as a limitation. Medical information is limited to the ICD-10 diagnoses recorded for the purposes of billing. These diagnoses are not generally audited and reflect to some extent the coding and clinical practices of the physicians. For example, we are unable to include patients whose diagnoses record only the symptoms experienced and not an underlying FSS. Additionally, we are unable to differentiate between a patient in remission and a patient who chooses no longer to consult for a persisting FSS. The data therefore provide the perspective of the ambulatory physicians, which is necessarily different from that provided by an epidemiological cohort such as the DanFunD study^28^.

Aside from the question of aetiology, it must be noted that the FSS exist within a complex, interacting bio-psychosocial context. Issues such as psychological comorbidity, healthcare-seeking behaviour, societal expectations and disease labelling are central to a phenomenological understanding of the syndromes^43^. While the present study highlights the biological and psychological aspects of the FSS, we are unable to observe the sociological aspects that may in part determine the expression and course of the disease.

## Conclusion

In conclusion, our study demonstrates the importance of the bio-psychosocial model both for an understanding of aetiology and for clinical practice. All FSS groups exhibit a wide range of common psychological and somatic complaints that are sustained over a period of more than five years both before and after first diagnosis of the condition. Further research is required into the role of biological triggers such as gastrointestinal infection and the opportunity they present for early intervention.

Patients with increasing physician contact rates should be identified by primary care physicians as patients at risk for developing FSS and screened for concomitant depression or anxiety. This might help to provide more timely support for these vulnerable patients and prevent such progression of the condition as was observed in the present cohort.

### Data sharing

The data that support the findings of this study are available from the Bavarian Association of Statutory Health Insurance Physicians but restrictions apply to the availability of these data, which were used under license for the current study, and so are not publicly available. Data are however available from the authors upon reasonable request and with permission of Bavarian Association of Statutory Health Insurance Physicians.

## Supplementary information

Supplementary information.
