# Supplementary material for: Comorbidities of Patients with Functional Somatic Syndromes Before, During and After First Diagnosis: A Population-based Study using Bavarian Routine Data
Source: Sci Rep. 2020 Jun 17;10:9810. doi: 10.1038/s41598-020-66685-4 (PMC7299983; doi:10.1038/s41598-020-66685-4)
Supplement: Supplementary file 1 — Supplementary information. [file 41598_2020_66685_MOESM1_ESM.pdf]

Comorbidities of Patients with Functional Somatic Syndromes Before, During and After First Diagnosis: A Population-based Study using Bavarian Routine Data

Supplementary Information

Ewan Donnachie Antonius Schneider Paul Enck

2020-03-12

Contents

1 List of ICD-10-GM Codes 2

2 Persistence of the FSS Diagnosis 3

3 Treatment Intensity for Various Diagnoses 6
3.1 Psychological Disorder . . . 6
3.2 General Symptoms . . . 10
3.3 Infection . . . 14
3.4 FSS . . . 18

1 List of ICD-10-GM Codes

The following table specifies the ICD-10-GM codes underlying the study. All codes are expanded to the right to encompass all subcodes (e.g. "M" includes all codes beginning with M, thus covering the whole of Chapter XIII of the ICD-10-GM). Only secured diagnoses are considered (i.e. codes marked "suspected", "exclusion of" or "condition following diagnosis" are ignored). All codes are specified for the year 2016; expansion and historisation was performed using the `ICD10gm` package for R (`https://cran.r-project.org/package=ICD10gm`).

Supplementary Table 1: ICD-10-Codes

Diagnosis	ICD-10-GM Code Specification
Irritable Bowel Syndrome (IBS)	F45.32, K58
Functional Intestinal Disorder (FID)	K59
Chronic Fatigue Syndrome (CFS)	F48.0, G93.3
Fibromyalgia Syndrome (FMS)	M79.7
Tension Headache (TH)	G44.2
Somatization Disorder (SD)	F45.0
Infectious Gastroenteritis	A01, A02, A03, A04.0, A04.1, A04.2, A04.3, A04.4, A04.5, A04.6, A04.7, A04.8, A04.9, A05, A06, A07, A08, A09.0
Non-specific Gastroenteritis	A09.9, K52.9
Upper respiratory infection	J01, J02, J03, J04, J05, J06
Any Infection	A1, A2, A3, A4, A5, A6, A7, A8, B, J1, J2
Psychological disorder	F32, F33, F41, F43
Anxiety	F06.4, F41, F60, F93.0, F93.1, F93.2
Depression	F32, F33, F92.0
Stress reaction disorder	F43
Psychosocial diagnosis	R45, R46, Z55, Z56, Z57, Z58, Z59, Z60, Z61, Z62, Z63, Z64, Z65, Z72, Z73
Fatigue	R53
Headache	R51, G44
Back pain	M54
Musculosceletal diseases	M

The functional somatic syndromes are abbreviated as follows:

- **CFS**: Chronic Fatigue Syndrome
- **IBS**: Irritable Bowel Syndrome
- **FID**: Functional Intestinal Disorder
- **FMS**: Fibromyalgia Syndrome
- **TH**: Tension Headache
- **SD**: Somatization Disorder
- **Multiple Diagnoses**: More than one of the above conditions in the quarter first diagnosis

2 Persistence of the FSS Diagnosis

The following tables report the proportion of patients whose diagnosis is present 1, 2, 3, 4 and 5 years following the initial diagnosis, as estimated using a Kaplan-Meier estimator. While the first table requires that the same diagnosis is present, the second table estimates considers patients transitioning to a different FSS group as having a persisting diagnosis.

Supplementary Table 2: Kaplan-Meier estimation for the time between first and last recorded diagnosis, considering both the persistence of the inital diagnosis and the record of any FSS.

Time	Diagnosis	Number of patients			% with persistent diagnosis
(years)	considered	at risk	last diagnosis	censored	(95% CI)
**CFS**					
1	Initial diagnosis	16305	676	0	77.4 (76.9, 78.0)
	Any FSS diagnosis	17085	558	0	81.9 (81.3, 82.4)
2	Initial diagnosis	13889	523	40	66.2 (65.6, 66.9)
	Any FSS diagnosis	15035	453	40	72.2 (71.6, 72.9)
3	Initial diagnosis	11831	440	25	57.0 (56.3, 57.7)
	Any FSS diagnosis	13199	409	27	64.0 (63.3, 64.6)
4	Initial diagnosis	10007	488	17	48.0 (47.4, 48.7)
	Any FSS diagnosis	11457	498	18	55.3 (54.6, 55.9)
5	Initial diagnosis	8092	465	17	38.8 (38.1, 39.5)
	Any FSS diagnosis	9518	481	18	45.9 (45.2, 46.6)
**IBS**					
1	Initial diagnosis	3498	148	0	76.2 (75.0, 77.5)
	Any FSS diagnosis	3801	103	0	84.2 (83.1, 85.2)
2	Initial diagnosis	3046	99	11	67.1 (65.7, 68.5)
	Any FSS diagnosis	3465	78	12	77.1 (75.8, 78.3)
3	Initial diagnosis	2614	78	7	58.4 (57.0, 59.9)
	Any FSS diagnosis	3083	77	7	69.2 (67.9, 70.6)
4	Initial diagnosis	2294	84	4	51.5 (50.0, 53.0)
	Any FSS diagnosis	2756	81	4	62.2 (60.8, 63.6)
5	Initial diagnosis	1963	86	9	44.1 (42.6, 45.6)
	Any FSS diagnosis	2390	98	9	53.7 (52.3, 55.2)
**FID**					
1	Initial diagnosis	4462	199	0	75.9 (74.8, 77.0)
	Any FSS diagnosis	4785	141	0	82.7 (81.7, 83.7)
2	Initial diagnosis	3753	154	14	64.1 (62.8, 65.3)
	Any FSS diagnosis	4241	127	14	73.2 (72.1, 74.4)
3	Initial diagnosis	3151	133	3	54.4 (53.1, 55.7)
	Any FSS diagnosis	3755	117	4	65.4 (64.2, 66.7)
4	Initial diagnosis	2630	138	5	45.1 (43.8, 46.4)
	Any FSS diagnosis	3269	130	5	56.8 (55.5, 58.1)
5	Initial diagnosis	2098	140	4	35.7 (34.4, 37.0)
	Any FSS diagnosis	2751	154	8	47.2 (45.9, 48.5)
**FMS**					
1	Initial diagnosis	639	12	0	87.1 (84.7, 89.6)
	Any FSS diagnosis	673	8	0	92.4 (90.4, 94.3)
2	Initial diagnosis	595	8	0	81.5 (78.7, 84.4)
	Any FSS diagnosis	641	7	0	88.1 (85.7, 90.5)
3	Initial diagnosis	554	11	2	76.0 (72.9, 79.1)
	Any FSS diagnosis	604	7	2	83.5 (80.8, 86.2)
4	Initial diagnosis	516	7	0	71.7 (68.5, 75.1)
	Any FSS diagnosis	574	7	0	79.8 (76.9, 82.8)
5	Initial diagnosis	469	5	0	66.1 (62.7, 69.6)
	Any FSS diagnosis	526	7	0	73.7 (70.6, 77.0)

**TH**					
1	Initial diagnosis	4799	255	0	71.1 (70.0, 72.2)
	Any FSS diagnosis	5277	198	0	79.4 (78.5, 80.4)
2	Initial diagnosis	3927	157	7	59.0 (57.8, 60.2)
	Any FSS diagnosis	4595	132	7	69.8 (68.7, 70.9)
3	Initial diagnosis	3323	129	6	50.4 (49.2, 51.7)
	Any FSS diagnosis	4075	130	6	62.2 (61.1, 63.4)
4	Initial diagnosis	2818	127	7	42.9 (41.7, 44.1)
	Any FSS diagnosis	3575	121	10	54.9 (53.7, 56.2)
5	Initial diagnosis	2279	108	4	34.9 (33.7, 36.1)
	Any FSS diagnosis	3010	134	5	46.2 (45.0, 47.4)
**SD**					
1	Initial diagnosis	4141	220	0	71.7 (70.5, 72.9)
	Any FSS diagnosis	4584	166	0	80.8 (79.8, 81.8)
2	Initial diagnosis	3369	151	13	58.9 (57.6, 60.2)
	Any FSS diagnosis	3994	121	15	70.8 (69.6, 72.0)
3	Initial diagnosis	2750	123	3	48.6 (47.3, 50.0)
	Any FSS diagnosis	3447	112	6	61.7 (60.4, 63.0)
4	Initial diagnosis	2276	97	7	40.7 (39.4, 42.0)
	Any FSS diagnosis	2994	106	7	53.9 (52.6, 55.2)
5	Initial diagnosis	1895	76	4	34.3 (33.0, 35.5)
	Any FSS diagnosis	2566	92	5	46.5 (45.2, 47.8)
**Multiple FSS**					
1	Initial diagnosis	760	22	0	82.2 (79.7, 84.7)
	Any FSS diagnosis	797	18	0	86.7 (84.6, 89.0)
2	Initial diagnosis	675	9	2	74.2 (71.4, 77.1)
	Any FSS diagnosis	723	9	2	79.5 (76.9, 82.2)
3	Initial diagnosis	606	20	0	66.3 (63.3, 69.5)
	Any FSS diagnosis	662	19	0	72.7 (69.8, 75.6)
4	Initial diagnosis	537	14	1	59.6 (56.5, 62.9)
	Any FSS diagnosis	588	14	1	65.4 (62.3, 68.6)
5	Initial diagnosis	475	14	1	53.0 (49.8, 56.3)
	Any FSS diagnosis	525	16	1	58.4 (55.3, 61.7)

Supplementary Table 3: Proportion of patients receiving a FSS diagnosis at any time during the five-year follow-up period.

	Proportion with Diagnosis During Follow-up (%)					
Leading Diagnosis	CFS	IBS	FID	FMS	TH	SD
Control	1.4	0.2	0.6	0.0	0.5	0.4
CFS	100.0	5.8	8.7	1.7	8.4	9.7
IBS	20.4	100.0	13.8	1.4	8.9	10.7
FID	20.1	12.3	100.0	1.3	7.9	7.6
FMS	25.8	6.9	10.3	100.0	9.9	15.0
TH	21.9	7.1	9.5	1.6	100.0	10.4
SD	22.7	7.2	10.2	2.6	9.5	100.0
Multiple FSS	74.8	32.7	34.7	10.6	35.3	54.5

3 Treatment Intensity for Various Diagnoses

The following supplementary figures extend Figure 4 to analyse further diagnoses of relevance to the study.

3.1 Psychological Disorder

3.1.1 Proportion in the Cohort

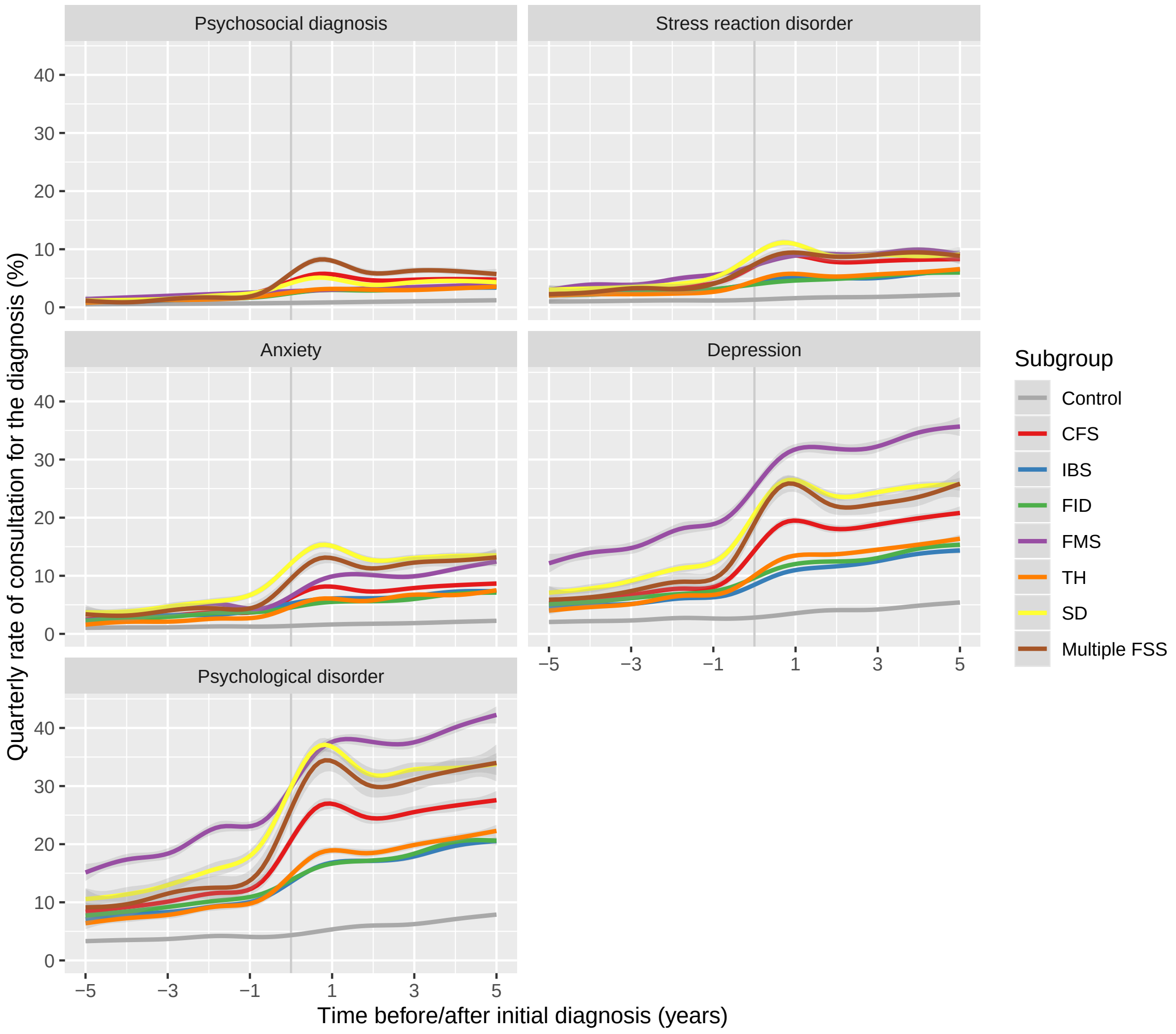

Supplementary Figure 1: Proportion of patients consulting in each quarterly period with psychological diagnoses (raw data without standardisation)

3.1.2 Adjusted by Age and Sex

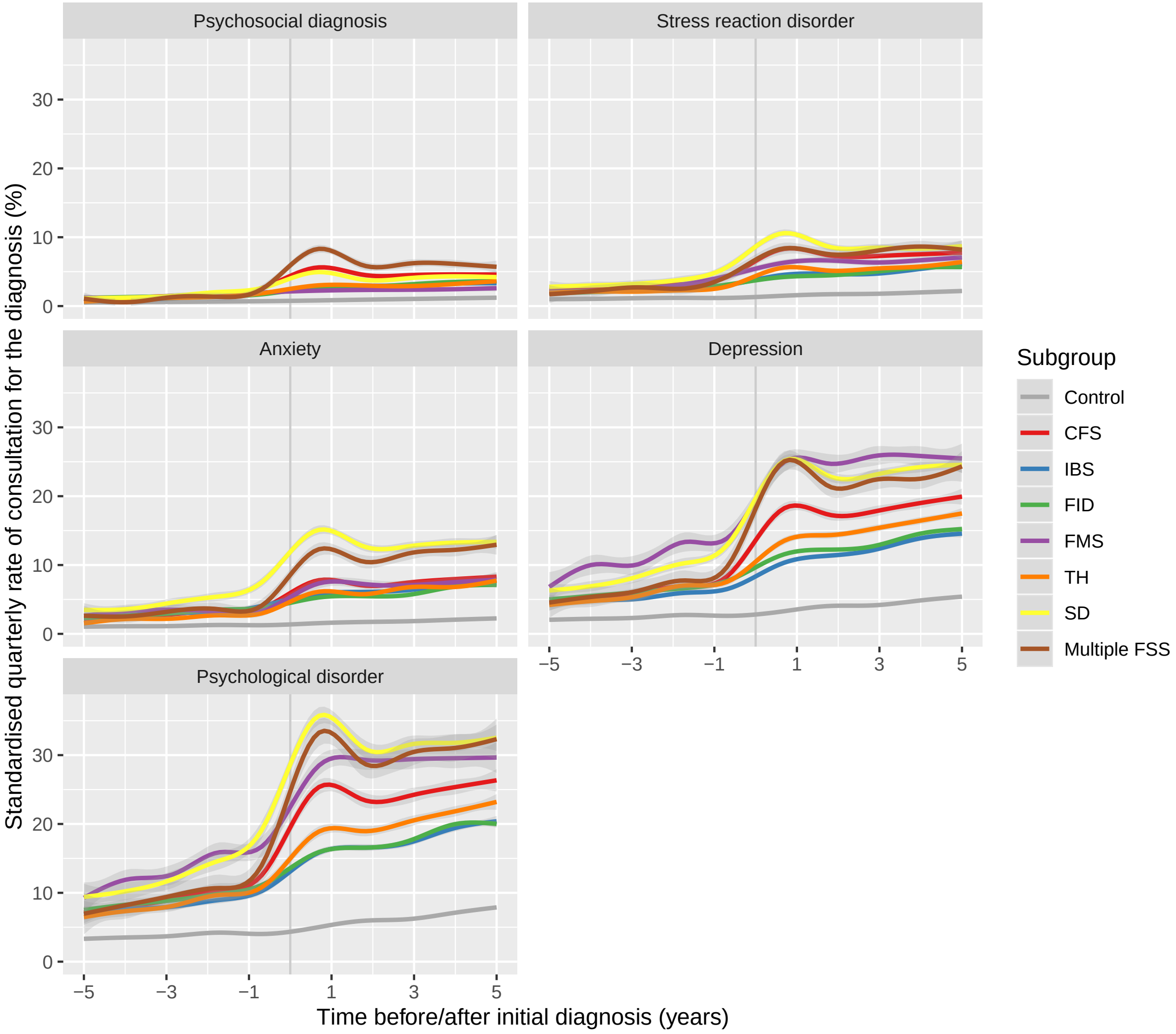

Supplementary Figure 2: Proportion of patients consulting in each quarterly period with psychological diagnoses (standardised by age and sex to the structure of the Bavarian population)

Supplementary Table 4: Psychological Comorbidity: Diagnoses before, concurrent with and after first diagnosis of a FSS, adjusted according to the age and sex distribution in the Bavarian population.

		Pre-incident			Incidence	Post-incident		
Diagnosis	Group	Any time prior	> 1 year prior	year prior		year post	> 1 year post	Any time post
Anxiety	Control	7.4	2.4	2.5	1.6	3.0	3.0	10.9
	CFS	17.2	6.3	7.4	9.3	13.9	12.4	33.7
	IBS	16.1	5.9	7.5	6.7	10.5	10.5	26.9
	FID	16.8	6.7	7.1	7.6	9.7	9.6	27.7
	FMS	15.1	5.9	6.9	7.4	13.8	12.9	30.9
	TH	14.7	5.0	6.1	7.0	10.7	10.4	28.9
	SD	24.4	10.0	13.8	18.1	23.7	19.4	42.4
	Multiple FSS	18.3	6.9	8.8	16.4	21.1	18.8	46.1
Depression	Control	13.0	5.0	5.1	3.7	6.4	6.8	20.2
	CFS	31.6	12.8	16.4	23.1	31.1	28.2	60.1
	IBS	24.2	10.5	11.7	10.9	17.7	18.0	41.3
	FID	26.3	11.2	13.5	12.1	19.2	19.3	43.1
	FMS	36.5	21.2	23.5	26.7	36.6	36.5	59.6
	TH	26.4	11.9	13.9	16.0	22.8	22.7	47.8
	SD	39.0	18.2	23.9	30.8	38.6	34.6	62.2
	Multiple FSS	30.4	13.9	17.0	29.7	40.4	34.0	62.8
Psychological disorder	Control	22.2	8.1	8.1	5.8	10.1	10.7	33.6
	CFS	46.6	19.6	24.5	33.9	42.1	38.5	75.6
	IBS	38.0	16.5	18.8	17.6	27.0	27.1	59.7
	FID	40.7	17.5	20.3	19.3	27.1	27.2	60.1
	FMS	43.4	24.7	27.8	31.4	42.9	44.8	69.1
	TH	39.9	17.5	20.1	24.0	31.8	31.1	64.7
	SD	54.3	26.2	34.5	46.1	52.8	46.3	77.8
	Multiple FSS	45.7	19.8	26.7	44.0	52.4	45.8	78.8

Psychosocial diagnosis	Control	7.3	1.8	2.0	1.1	2.3	2.6	13.3
	CFS	18.1	5.0	7.1	11.5	13.7	11.3	39.6
	IBS	13.9	4.0	4.9	3.8	7.3	6.8	27.0
	FID	14.6	3.9	4.6	3.4	7.1	6.8	28.4
	FMS	16.5	4.5	5.0	3.3	5.9	6.7	26.3
	TH	13.6	4.0	4.9	4.7	7.3	7.6	29.2
	SD	18.3	5.3	7.4	8.2	10.9	8.8	33.9
	Multiple FSS	16.1	4.3	6.5	13.6	16.4	13.4	39.7
Stress reaction disorder	Control	10.9	2.9	2.9	1.8	3.7	4.0	18.9
	CFS	25.4	7.8	9.8	12.5	17.3	15.5	48.1
	IBS	19.1	6.0	6.8	5.6	9.9	10.0	36.4
	FID	19.5	5.6	6.8	4.7	9.7	9.7	35.6
	FMS	22.8	5.8	9.4	6.9	12.0	13.5	37.0
	TH	19.9	5.7	6.8	8.4	12.0	11.2	38.5
	SD	28.0	8.9	12.9	16.1	20.1	16.9	47.8
	Multiple FSS	24.3	6.7	10.7	14.2	20.1	14.5	46.1

3.2 General Symptoms

3.2.1 Proportion in the Cohort

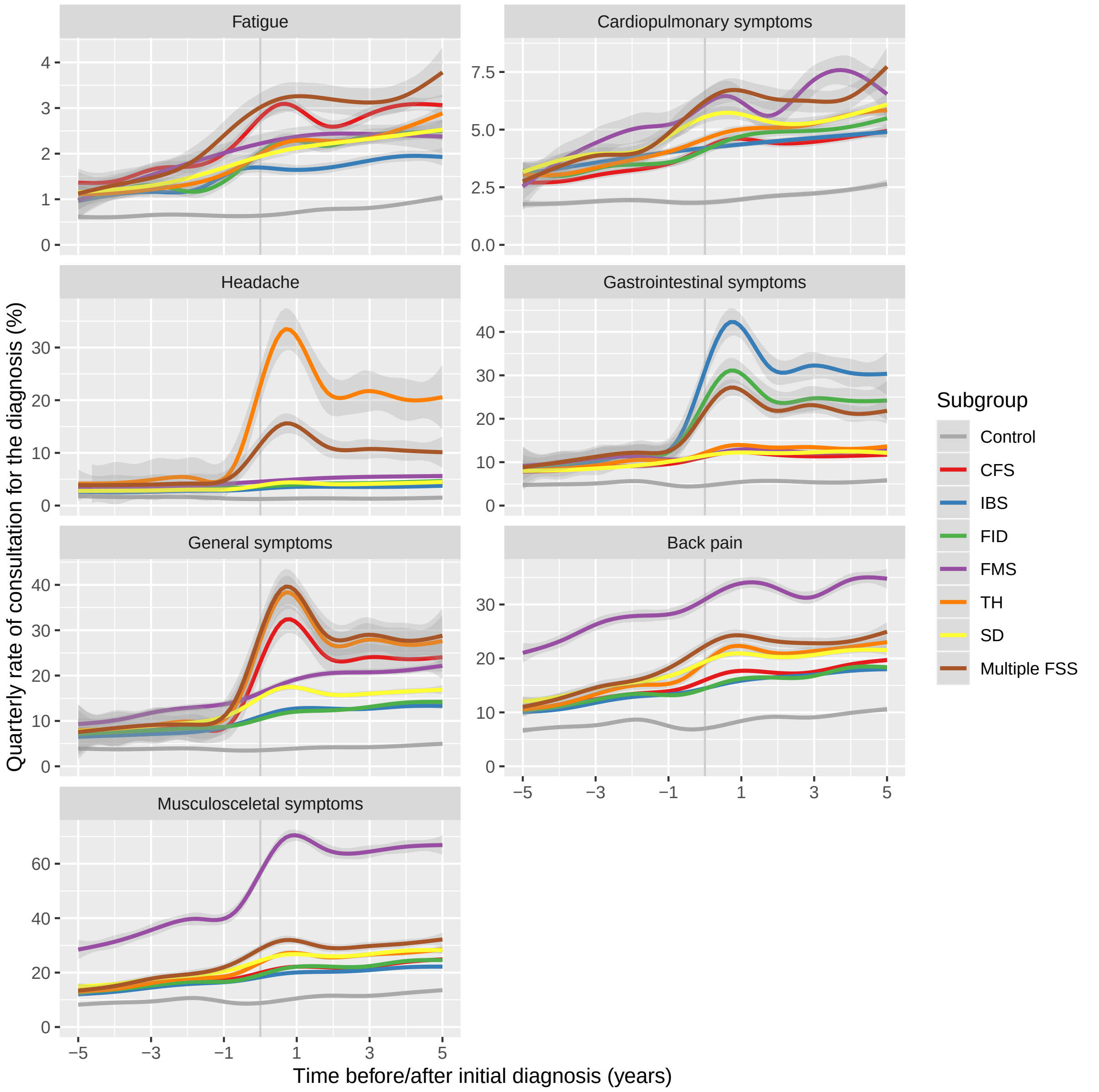

Supplementary Figure 3: Proportion of patients consulting in each quarterly period with general symptoms (raw data without standardisation)

3.2.2 Adjusted by Age and Sex

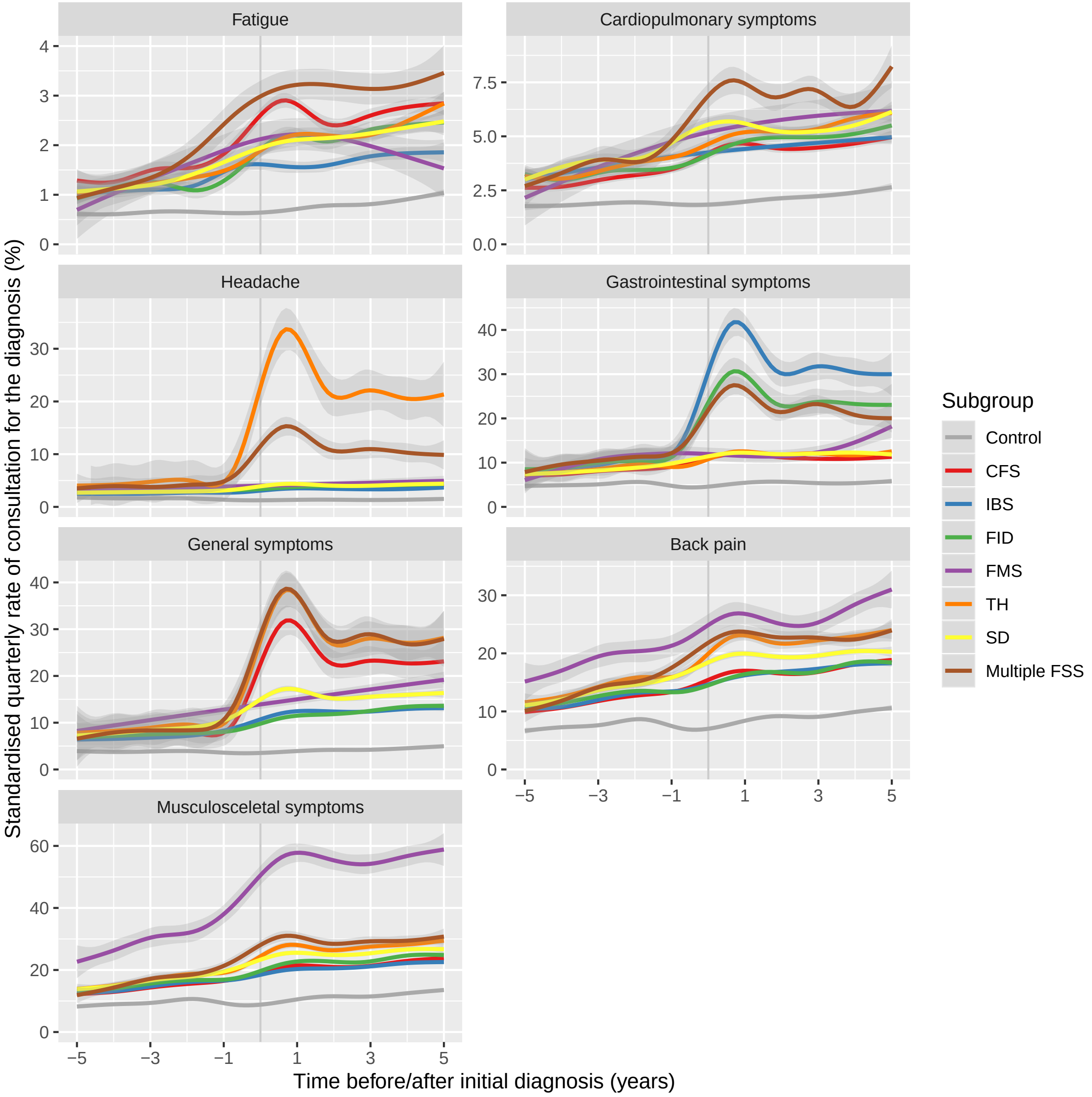

Supplementary Figure 4: Proportion of patients consulting in each quarterly period with general symptoms (standardised by age and sex to the structure of the Bavarian population)

Supplementary Table 5: General Symptoms: Diagnoses before, concurrent with and after first diagnosis of a FSS, adjusted according to the age and sex distribution in the Bavarian population.

		Pre-incident			Incidence	Post-incident		
Diagnosis	Group	Any time prior	> 1 year prior	year prior		year post	> 1 year post	Any time post
Cardiopulmonary symptoms	Control	23.7	6.0	5.5	2.9	5.9	6.3	30.6
	CFS	35.2	9.9	10.4	8.3	12.8	12.5	47.2
	IBS	37.3	11.3	12.4	5.7	11.5	12.4	46.6
	FID	36.1	10.0	10.3	6.8	12.9	12.8	49.2
	FMS	40.8	10.7	16.2	7.4	16.4	10.8	46.2
	TH	36.7	10.9	11.5	6.8	13.5	13.7	49.2
	SD	41.6	11.9	14.6	9.8	15.3	13.6	50.5
	Multiple FSS	39.4	10.8	15.1	12.2	17.2	15.0	51.0
Gastrointestinal symptoms	Control	50.3	17.2	13.9	8.9	17.1	17.7	59.3
	CFS	67.5	25.5	28.4	17.4	32.8	31.8	78.6
	IBS	74.9	32.0	40.1	100.2	79.7	61.9	100.2
	FID	71.5	30.3	34.0	100.1	69.6	54.8	100.1
	FMS	73.7	26.4	37.9	12.9	33.4	27.0	79.6
	TH	68.0	26.9	27.0	14.2	32.5	33.0	79.7
	SD	68.1	25.9	29.2	18.4	32.9	32.2	78.2
	Multiple FSS	75.7	32.0	37.2	51.3	55.2	48.7	89.1
General symptoms	Control	36.9	10.6	9.8	5.2	11.1	11.3	43.8
	CFS	59.1	20.9	24.4	100.1	67.9	50.8	100.1
	IBS	55.5	19.5	21.8	17.0	28.1	27.9	71.3
	FID	56.0	20.0	21.0	14.2	27.3	26.4	70.1
	FMS	68.6	22.0	32.5	16.7	34.5	29.5	77.8
	TH	64.5	24.2	30.3	100.1	76.0	57.0	100.1
	SD	62.5	23.5	28.5	27.3	37.2	33.3	77.4
	Multiple FSS	61.9	21.4	30.9	82.5	71.1	55.9	95.1

Back pain	Control	51.8	20.8	17.9	11.4	20.8	22.1	61.9
	CFS	67.7	30.1	32.3	23.0	36.8	36.0	79.3
	IBS	67.0	31.2	31.1	18.1	34.2	35.8	77.5
	FID	66.0	30.9	30.1	17.7	34.8	35.3	76.9
	FMS	78.7	36.7	46.3	40.4	52.9	45.7	90.8
	TH	71.3	34.9	36.2	35.1	47.6	44.6	85.5
	SD	69.2	33.3	35.5	26.9	40.6	38.9	79.4
	Multiple FSS	71.0	33.8	39.2	32.3	45.8	44.1	81.4
Fatigue	Control	9.5	2.1	2.0	1.0	2.3	2.6	14.9
	CFS	20.0	5.1	6.4	6.5	8.5	7.3	33.3
	IBS	17.0	3.8	5.2	3.1	4.9	4.8	25.7
	FID	16.7	3.7	4.5	3.7	6.7	6.3	28.6
	FMS	18.0	3.7	6.8	2.4	7.6	6.3	24.0
	TH	17.1	4.2	4.8	3.2	6.4	6.4	29.5
	SD	18.6	4.5	5.6	4.4	6.4	5.7	28.9
	Multiple FSS	19.6	4.4	7.3	7.1	8.3	8.5	31.6
Headache	Control	19.6	4.8	4.0	1.9	4.3	4.2	19.6
	CFS	31.8	8.8	9.1	6.7	11.1	10.9	38.4
	IBS	28.7	8.2	7.7	3.9	9.4	9.7	34.6
	FID	32.2	9.5	9.2	4.6	10.5	10.4	37.5
	FMS	41.0	6.7	10.1	4.6	11.4	9.3	40.4
	TH	45.5	13.6	18.8	100.1	71.1	47.6	100.1
	SD	32.9	9.4	9.2	6.8	11.9	11.1	38.3
	Multiple FSS	35.9	12.0	13.2	33.1	30.9	23.3	58.0
Musculosceletal symptoms	Control	60.9	25.6	22.3	14.3	25.9	27.5	72.0
	CFS	76.2	36.5	39.4	29.0	45.0	44.5	87.4
	IBS	75.9	37.4	38.0	23.2	42.6	42.7	85.9
	FID	74.9	37.6	38.4	28.6	46.9	46.2	87.6
	FMS	93.6	54.6	71.3	100.0	85.9	82.9	100.0
	TH	78.6	40.9	44.0	42.3	56.2	52.6	91.2
	SD	77.6	39.7	43.2	33.7	49.4	48.2	87.3
	Multiple FSS	79.4	40.5	47.2	44.8	57.5	52.9	90.6

3.3 Infection

3.3.1 Proportion in the Cohort

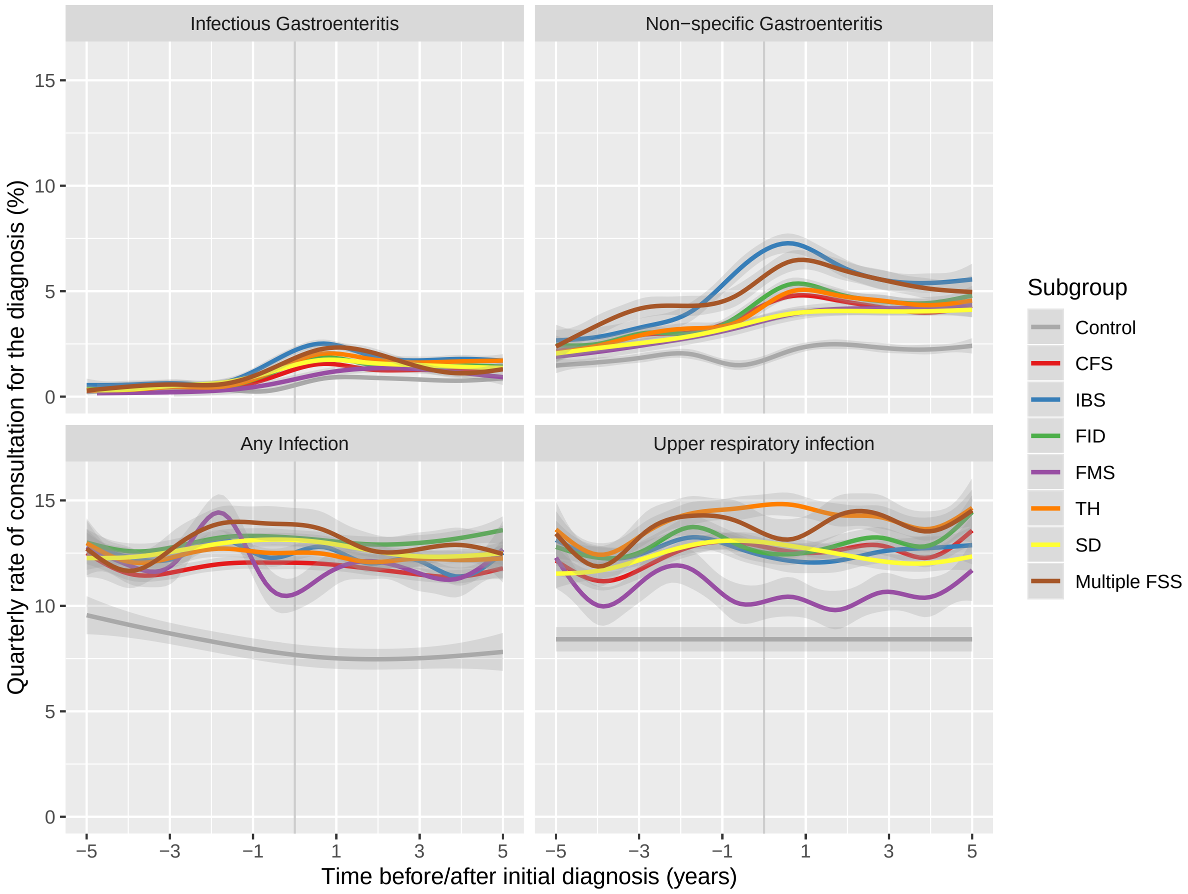

Supplementary Figure 5: Proportion of patients consulting in each quarterly period with infection (raw data without standardisation)

3.3.2 Adjusted by Age and Sex

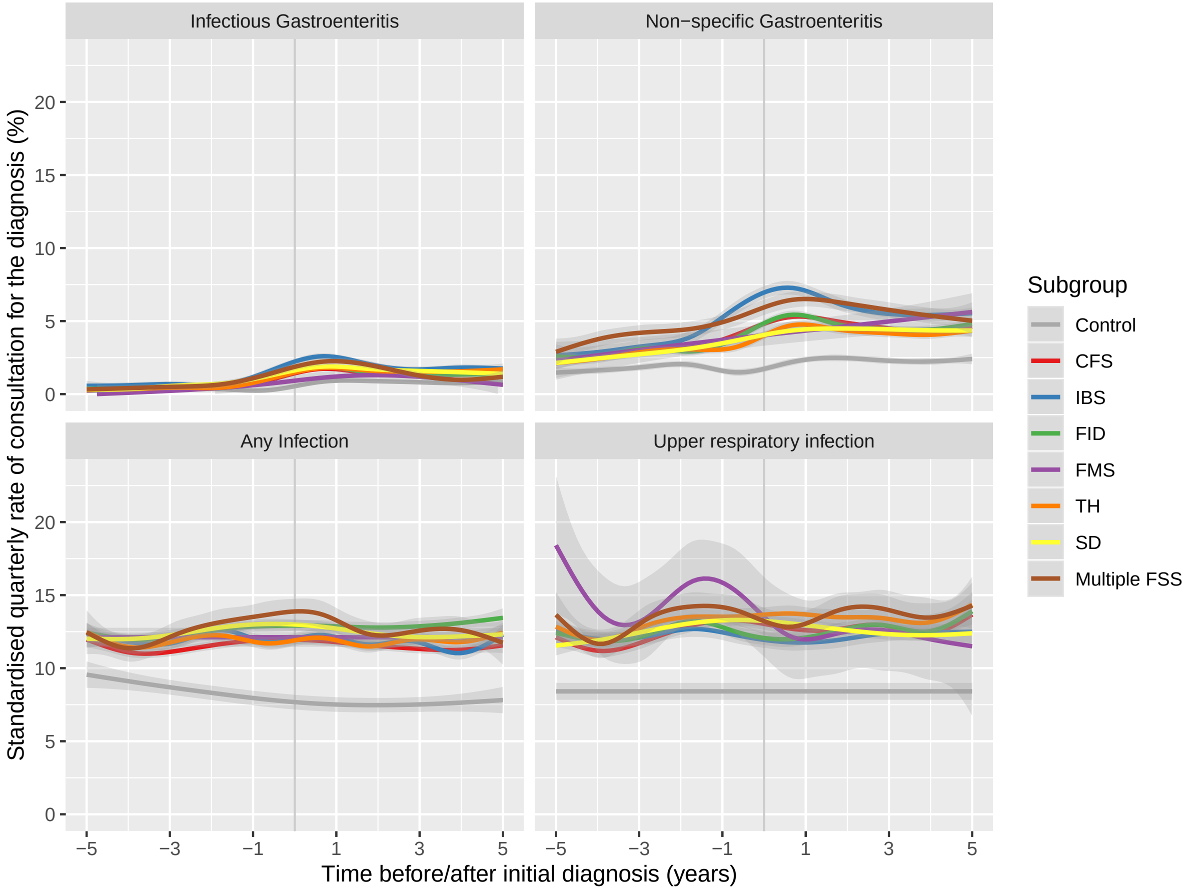

Supplementary Figure 6: Proportion of patients consulting in each quarterly period with infection (standardised by age and sex to the structure of the Bavarian population)

Supplementary Table 6: Infection: Diagnoses before, concurrent with and after first diagnosis of a FSS, adjusted according to the age and sex distribution in the Bavarian population.

		Pre-incident			Incidence	Post-incident		
Diagnosis	Group	Any time prior	> 1 year prior	year prior		year post	> 1 year post	Any time post
Infectious Gastroenteritis	Control	4.7	1.2	1.0	1.6	3.2	3.1	14.9
	CFS	9.2	1.8	3.5	2.3	5.9	5.1	22.3
	IBS	11.4	2.3	5.4	5.4	8.3	6.6	25.9
	FID	9.2	1.8	3.8	3.0	6.1	5.6	21.8
	FMS	6.1	2.0	1.7	1.8	6.6	3.4	20.6
	TH	8.7	1.7	3.7	2.3	5.9	5.5	23.2
	SD	9.9	2.3	4.1	3.3	6.5	5.8	23.8
	Multiple FSS	9.8	2.1	4.3	3.5	7.3	7.0	22.9
Non-specific Gastroenteritis	Control	24.0	6.8	5.0	4.3	8.0	8.4	34.4
	CFS	38.1	11.1	13.6	7.3	16.5	16.0	51.9
	IBS	41.7	12.5	18.1	18.7	21.4	17.7	54.6
	FID	36.8	10.1	12.6	10.0	15.9	15.3	50.2
	FMS	39.9	7.0	19.6	4.9	14.1	9.4	49.0
	TH	35.1	10.1	10.8	5.1	14.5	14.1	48.6
	SD	35.7	10.3	12.2	6.2	14.3	13.8	48.6
	Multiple FSS	44.9	14.1	15.8	14.6	20.2	20.2	54.6
Any Infection	Control	68.9	25.4	22.0	11.4	22.2	21.5	67.2
	CFS	79.6	32.5	33.7	15.4	32.3	31.0	79.5
	IBS	80.4	34.5	32.5	15.4	33.9	32.0	81.0
	FID	80.3	35.0	34.4	16.2	34.2	32.4	82.2
	FMS	70.9	31.9	27.3	12.9	30.2	35.1	82.6
	TH	79.6	33.1	32.4	14.1	33.6	30.9	80.0
	SD	80.7	35.1	35.1	16.9	34.3	32.4	81.0
	Multiple FSS	79.5	34.0	35.5	18.6	35.4	30.6	81.4

Upper respiratory infection	Control	70.0	28.0	24.0	12.9	24.8	24.6	72.2
	CFS	81.2	37.8	38.5	16.1	37.1	36.2	85.0
	IBS	81.2	36.6	35.4	13.5	34.2	33.8	83.0
	FID	80.8	36.6	36.8	13.2	35.0	34.7	82.5
	FMS	80.5	40.9	37.6	11.0	30.1	35.5	81.3
	TH	82.8	38.9	38.6	17.5	39.0	37.7	85.6
	SD	81.2	37.5	37.1	17.0	36.8	35.5	82.6
	Multiple FSS	84.1	39.6	38.5	16.3	37.3	37.7	85.3

3.4 FSS

3.4.1 Proportion in the Cohort

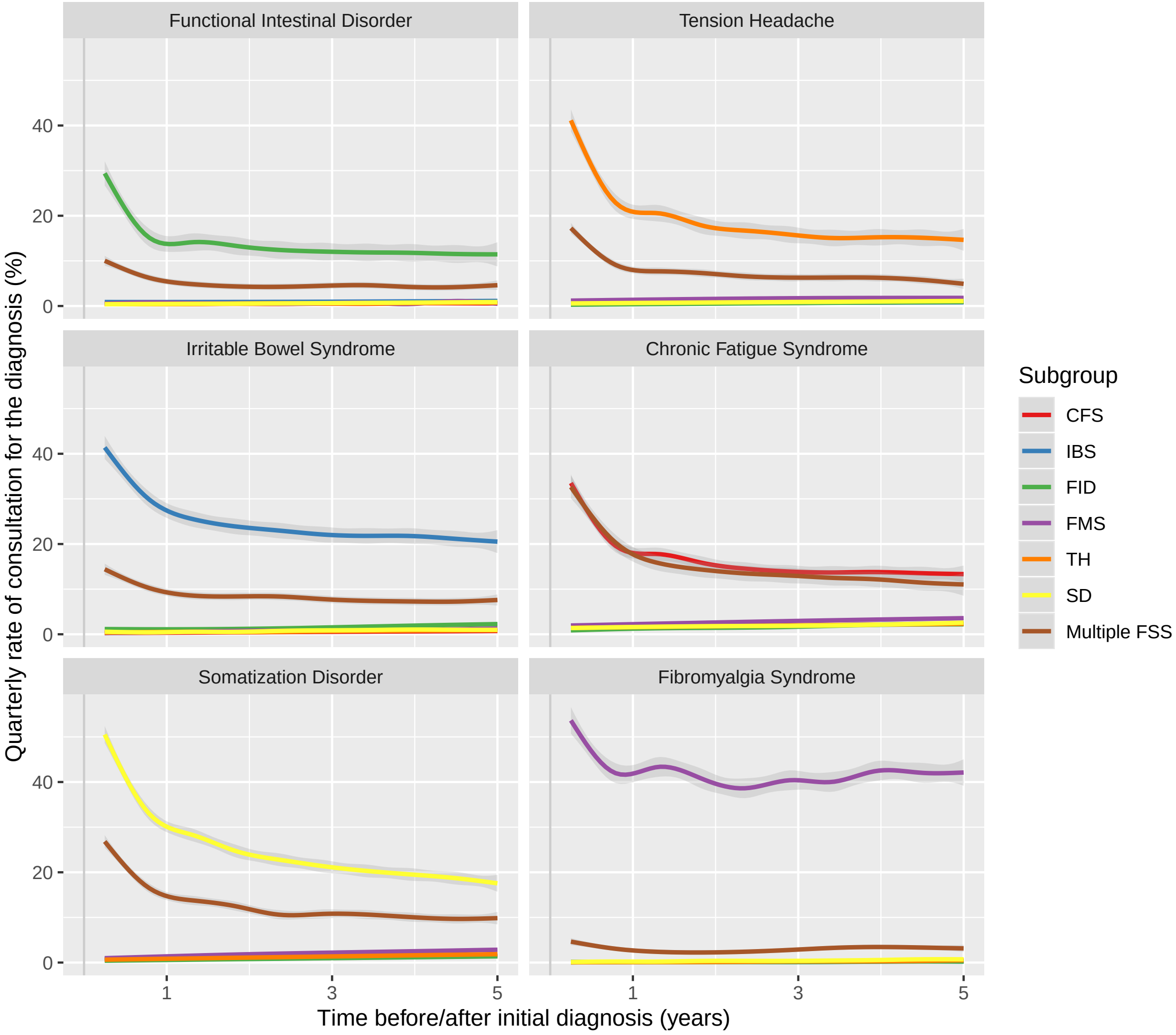

Supplementary Figure 7: Proportion of patients consulting in each quarterly period with functional somatic syndromes (raw data without standardisation)

3.4.2 Adjusted by Age and Sex

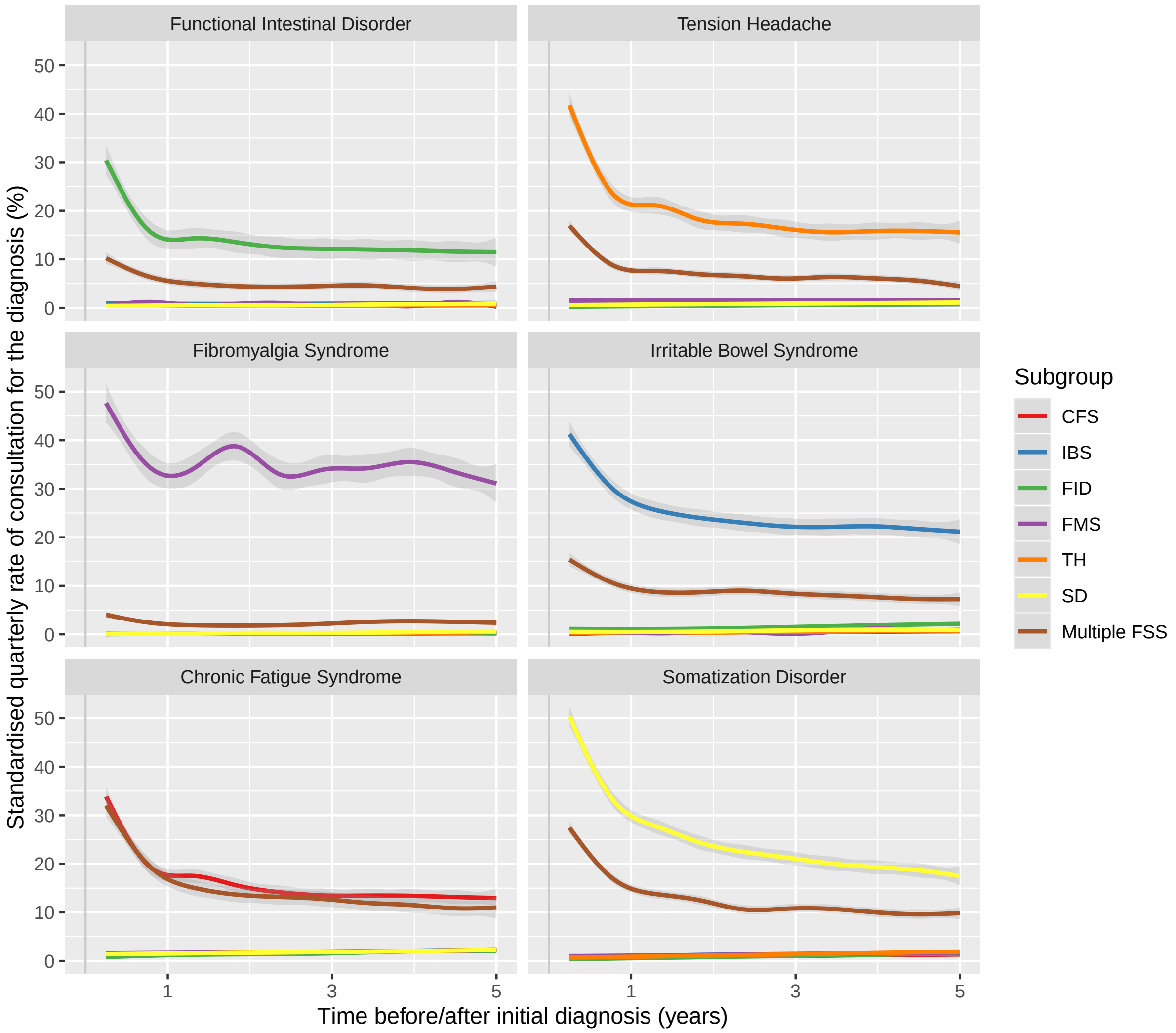

Supplementary Figure 8: Proportion of patients consulting in each quarterly period with functional somatic syndromes (standardised by age and sex to the structure of the Bavarian population)

Supplementary Table 7: Functional Somatic Syndromes: Diagnoses before, concurrent with and after first diagnosis of a FSS, adjusted according to the age and sex distribution in the Bavarian population.

		Pre-incident			Incidence	Post-incident		
Diagnosis	Group	Any time prior	> 1 year prior	year prior		year post	> 1 year post	Any time post
Chronic Fatigue Syndrome	Control	0	0	0	0.0	0.0	0.0	1.4
	CFS	0	0	0	100.1	56.3	34.2	100.1
	IBS	0	0	0	1.0	3.3	3.9	19.5
	FID	0	0	0	0.4	3.3	3.6	18.8
	FMS	0	0	0	1.1	5.9	3.6	21.6
	TH	0	0	0	1.0	4.1	4.4	20.8
	SD	0	0	0	1.6	4.1	4.6	21.9
	Multiple FSS	0	0	0	65.2	47.8	27.5	73.7
Fibromyalgia Syndrome	Control	0	0	0	0.0	0.0	0.0	0.0
	CFS	0	0	0	0.0	0.2	0.3	1.3
	IBS	0	0	0	0.0	0.3	0.3	1.3
	FID	0	0	0	0.0	0.2	0.2	1.1
	FMS	0	0	0	100.0	71.3	60.9	100.0
	TH	0	0	0	0.0	0.1	0.2	1.5
	SD	0	0	0	0.0	0.3	0.5	2.2
	Multiple FSS	0	0	0	6.7	6.0	3.2	8.8
Functional Intestinal Disorder	Control	0	0	0	0.0	0.0	0.0	0.6
	CFS	0	0	0	0.3	1.3	1.4	8.1
	IBS	0	0	0	2.2	2.8	2.6	13.1
	FID	0	0	0	100.1	51.7	31.3	100.1
	FMS	0	0	0	0.0	2.8	1.7	11.4
	TH	0	0	0	0.3	1.5	1.4	8.8
	SD	0	0	0	0.4	1.6	1.8	10.0
	Multiple FSS	0	0	0	25.9	16.8	10.0	34.6

Irritable Bowel Syndrome	Control	0	0	0	0.0	0.0	0.0	0.2
	CFS	0	0	0	0.3	0.9	1.1	5.4
	IBS	0	0	0	100.2	68.4	44.4	100.2
	FID	0	0	0	1.4	3.2	2.6	11.5
	FMS	0	0	0	0.4	0.7	0.8	4.7
	TH	0	0	0	0.1	1.0	1.1	6.2
	SD	0	0	0	0.4	1.4	1.3	7.0
	Multiple FSS	0	0	0	28.6	23.0	14.8	33.9
Somatization Disorder	Control	0	0	0	0.0	0.0	0.0	0.4
	CFS	0	0	0	0.5	1.8	1.9	9.4
	IBS	0	0	0	0.2	2.1	2.5	10.7
	FID	0	0	0	0.2	1.1	1.7	7.5
	FMS	0	0	0	0.4	3.2	3.0	12.0
	TH	0	0	0	0.5	2.0	2.3	10.6
	SD	0	0	0	100.2	74.2	45.8	100.2
	Multiple FSS	0	0	0	48.3	39.6	22.4	55.3
Tension Headache	Control	0	0	0	0.0	0.0	0.0	0.5
	CFS	0	0	0	0.5	1.4	1.5	7.8
	IBS	0	0	0	0.3	1.5	1.7	8.1
	FID	0	0	0	0.3	1.0	1.2	6.9
	FMS	0	0	0	0.1	3.8	2.0	9.2
	TH	0	0	0	100.1	64.8	37.9	100.1
	SD	0	0	0	0.5	1.9	2.1	9.2
	Multiple FSS	0	0	0	28.7	22.9	14.1	34.8
